# Divergent DNA Methylation Provides Insights into the Evolution of Duplicate Genes in Zebrafish

**DOI:** 10.1534/g3.116.032243

**Published:** 2016-09-19

**Authors:** Zaixuan Zhong, Kang Du, Qian Yu, Yong E. Zhang, Shunping He

**Affiliations:** * The Key Laboratory of Aquatic Biodiversity and Conservation of Chinese Academy of Sciences, Institute of Hydrobiology, Chinese Academy of Sciences, Wuhan 430072, Hubei, People’s Republic of China; † Institute of Hydrobiology, University of Chinese Academy of Sciences, Beijing 100039, People’s Republic of China; ‡ Key Laboratory of the Zoological Systematic and Evolution, Institute of Zoology, Beijing 100000, People’s Republic of China; § State Key Laboratory of Integrated Management of Pest Insects and Rodents, Institute of Zoology, Beijing 100000, People’s Republic of China

**Keywords:** divergence, duplication, methylation, transcriptome, zebrafish

## Abstract

The evolutionary mechanism, fate and function of duplicate genes in various taxa have been widely studied; however, the mechanism underlying the maintenance and divergence of duplicate genes in *Danio rerio* remains largely unexplored. Whether and how the divergence of DNA methylation between duplicate pairs is associated with gene expression and evolutionary time are poorly understood. In this study, by analyzing bisulfite sequencing (BS-seq) and RNA-seq datasets from public data, we demonstrated that DNA methylation played a critical role in duplicate gene evolution in zebrafish. Initially, we found promoter methylation of duplicate genes generally decreased with evolutionary time as measured by synonymous substitution rate between paralogous duplicates (Ks). Importantly, promoter methylation of duplicate genes was negatively correlated with gene expression. Interestingly, for 665 duplicate gene pairs, one gene was consistently promoter methylated, while the other was unmethylated across nine different datasets we studied. Moreover, one motif enriched in promoter methylated duplicate genes tended to be bound by the transcription repression factor FOXD3, whereas a motif enriched in the promoter unmethylated sequences interacted with the transcription activator Sp1, indicating a complex interaction between the genomic environment and epigenome. Besides, body-methylated genes showed longer length than body-unmethylated genes. Overall, our results suggest that DNA methylation is highly important in the differential expression and evolution of duplicate genes in zebrafish.

Gene duplication, which occurs in almost all types of life forms (Kondrashov *et al.* 2002), is the main source of evolutionary novelty (Ohno 1970) and morphological complexity (Freeling and Thomas 2006). Most teleost, including *Danio rerio*, experienced genome duplication three times, with the most recent genome duplication dating to 320–400 MYA (Van de Peer and Meyer 2003; Jaillon *et al.* 2004; Kasahara *et al.* 2007; Hoegg *et al.* 2004). The exceptions are common carp and rainbow trout (Xu *et al.* 2014; Berthelot *et al.* 2014), both of which have undergone a fourth duplication. Several models of the emergence, maintenance, and evolution of duplicate gene copies have been proposed (Innan and Kondrashov 2010). Duplicate genes can be preserved through subfunctionalization, neofunctionalization, and dosage selection (Conant and Wolfe 2008; Hahn 2009). Nucleotide substitution, *cis*-regulation, and epigenetic modifications, influence the expression and functional evolution of duplicate genes (Hahn 2009; Betran *et al.* 2006; Wang *et al.* 2014; Chang and Liao 2012).

DNA methylation, an epigenetic DNA modification that occurs at cytosine residues, is involved in various important biological processes, such as the regulation of repetitive element expression, the development of early embryogenesis, cell type differentiation, genomic imprinting, and X-inactivation (Bird 2002; Bestor 2000; Smith *et al.* 2015; Edwards and Ferguson-Smith 2007). Promoter methylation is often associated with transcription repression, whereas intragenic methylation likely controls expression from alternative promoter regions and hinders transcription elongation (Suzuki and Bird 2008; Maunakea *et al.* 2010; Bell and Felsenfeld 2000; Kass *et al.* 1997). Notably, epigenetic silencing of duplicates may aid in functional divergence (Rodin and Riggs 2003), and DNA methylation patterns play an important role in duplicate gene evolution (Widman *et al.* 2009; Keller and Yi 2014; Feng *et al.* 2010).

Previous study in humans demonstrated that DNA methylation exhibits striking degrees of evolutionary conservation (Keller and Yi 2014). DNA methylation divergence of duplicate genes is significantly correlated with gene expression divergence (Keller and Yi 2014). Duplicate genes show highly consistent patterns of DNA methylation divergence across multiple tissues due to different frequency of motifs (Keller and Yi 2014). Since zebrafish has been subjected to one more round whole genome duplication (WGD) compared to humans, we wondered whether the aforementioned patterns of DNA methylation of duplicate genes in human were different in zebrafish. In this study, we investigated the relationship between duplicate gene evolution and DNA methylation divergence. For example, we observed how the methylation level changed with evolutionary time (Ks), whether gene expression was coupled with DNA methylation, and how methylation divergence contributed to expression divergence. Since DNA methylation is important for early embryogenesis (Li *et al.* 1992), we also investigated DNA methylation patterns of duplicate genes during early developmental stages. All these results provided an answer to how DNA methylation influenced evolution of duplicate genes in zebrafish.

## Materials and Methods

### Identification of duplicate genes

All the corresponding nucleotide and protein sequences of zebrafish were retrieved from Ensembl (http://asia.ensembl.org/index.html). To search potential zebrafish duplicate gene pairs, we initially used BLASTP (Altschul *et al.* 1997) with default parameters. Briefly, each protein sequence was compared against every other protein sequence in the zebrafish genome. Our criteria for whether two genes were considered a gene pair were proposed by Gu *et al.* (2002): (1) the alignable region between the two protein sequences should be longer than 80% of the longer region; (2) the identity between the two sequences (I) should be *I* ≥ 30% if the alignable region is longer than 150 amino acids, and *I* ≥ 0.01 *n* + 4.8 *L*^−0.32[1 + exp(−^*^L^*^/1000)]^ for all other proteins, where *n* = 6, and *L* is the alignable length between the two proteins (Rost 1999). Based on these initial pairings, gene families were created by performing the Markov Cluster Algorithm (http://micans.org/mcl/) until no additional groups shared a member. For each gene family, we aligned the protein sequences using MUSCLE (Edgar 2004). Using the yn00 module in PAML (Yang 2007), we calculated Ks pairwise, and selected the gene pair with the lowest Ks. We calculated the ratio of nonsynonymous to synonymous substitutions per site (Ka/Ks) of these duplicate genes using PAML4 (Yang 2007) to examine the functional constraints. A Ka/Ks ratio (ω) > 1 indicated positive selection, whereas a ratio < 1 indicated functional constrain. An LRT was conducted to determine whether Ka/Ks between the duplicate pairs was significantly lower than 0.5 (Zou *et al.* 2012; J. Wang *et al.* 2013). The Codeml program or PAML4 was run twice [model = 0 (fixing ω = 0.5), and model = 1] for each pair. Then twice the log likelihood difference of these two runs was compared to a Chi-square distribution with df = 1 (Yang 1998). The false discovery rates (FDR) were controlled using the Benjamini-Hochberg method (Klipper-Aurbach *et al.* 1995) with an FDR of 5%. ω < 0.5 (*P* < 0.01) may indicate evolutionary constraint.

### Comparing the functional domains of duplicate genes

We used Interproscan (Jones *et al.* 2014) to identify the domains for duplicate genes by scanning the protein domains and important sites to determine any potential functions. The functional divergence of the duplicate copies was detected by comparing the domains. We attributed two genes to the same functional group if they contained the same domains. The pairs that had distinct domains belonged to different functional groups.

### Analysis of DNA methylation data

Methylation data for egg, sperm, testis, and six stages (16-cell, 32-cell, 64-cell, 128-cell, 1k-cell, and germ ring), were obtained from NCBI with accession number PRJNA188516, which used Bisulfite sequencing (BS-seq). Genomic DNA (R100 ng) spiked with 0.5% unmethylated cl857 Sam7 Lambda DNA (Promega) was used to construct the DNA library provided a measure of the sum of the rates of nonconversion, and thymidine to cytosine-sequencing errors (Jiang *et al.* 2013). The Zv9 reference genome was downloaded from Ensembl (http://asia.ensembl.org/index.html). Trimmomatic was performed to trim the reads with default parameters. We mapped the filtered paired-end reads against the reference genome using Bismark_v0.13.0 (Krueger and Andrews 2011) with the following stringent parameters: –n 2 –l 60 –e 100 –X 600. A promoter was defined as 2 kb upstream from the transcriptional start site, and the gene body comprised the remainder of the gene region. On one hand, we estimated methylation level as *m*_i_/(*m*_i_ + *u*_i_), which represents the probability that CpG *i* is methylated in a sample (Jiang *et al.* 2013).

In addition, we applied another way to evaluate a region was methylated or unmethylated (Takuno and Gaut 2012, 2013). The methylation level of CpGs was calculated by

$$P_{\mathrm{CG}}=\sum_{i=m_{\mathrm{cg}}}^{n_{\mathrm{cg}}}\left(\begin{array}{c} n_{\mathrm{cg}}\\ i \end{array}\right)p_{\mathrm{cg}}^{i}{\left(1-p_{\mathrm{cg}}\right)}^{n_{\mathrm{cg}}-i}$$

where *P*_CG_ is a proxy of DNA methylation level (Takuno and Gaut 2012, 2013). *p*_cg_ is the proportion of methylated cytosine residues at CpG sites across the whole genome. *n*_cg_ and *m*_cg_ represents the number of cytosine residues at CpG sites with >2 coverage, and the number of methylated cytosine residues at CpG sites in a gene, respectively. We kept only those genes with sufficient CpG information (*n*_cg_ ≥ 20) and genes for which 60% of cytosine residues were covered by at least two reads (Takuno and Gaut 2012). When the *P*_CG_ value is low, the region was more densely methylated than expected at random. We use *P*_CG_ to define the region is methylated or unmethylated using the criteria of *P*_CG_ ≤ 0.05 or *P*_CG_ ≥ 0.95, respectively.

### RNA-seq data analysis

Available paired end FASTQ sequence files for sperm, egg, 1000 cells, and germ ring were obtained from NCBI with accession number PRJNA188516 (Jiang *et al.* 2013). RNA-seq data of 16-cell, 32-cell, and 128-cell were downloaded with accession number PRJNA127881 (Aanes *et al.* 2011). RNA-seq data of testis was obtained with SRA number SRR1695730. Each read was separately mapped against Danio rerio Zv9 references (http://asia.ensembl.org/info/data/ftp/index.html) using the software Tophat (Trapnell *et al.* 2009, 2012). Reads that were longer than 48, and had no more than one multihit, were retained for next procedure. Considering that the high sequence similarity of duplicated genes might lead to the multiple alignment of sequencing reads, read counts used in expression analysis was based on a subset of uniquely aligned reads. For each gene, the normalized expression level was measured by fragments per kilobase of exon per million fragments mapped (FPKM) using Cufflinks (Trapnell *et al.* 2012).

To evaluate expression specificity of promoter methylated and unmethylated genes, we calculated *H*(*g*), the Shannon entropy, which is expressed in bits of the expression the vector of gene *g*. This practice is based on FPKM. The specificity score was defined as 1 − *H*(*g*) / log_2_(*N*), where *N* represents the number of points in time or the types of tissue (Pauli *et al.* 2012).

$$H\left(g\right)\mathrm{=-}\sum_{i=\text{1}}^{Ν}P_{i}\log_{2}P_{i}\;P_{i}=g_{i}/g_{\mathrm{sum}}$$

where *g* is the gene name; *g_i_* is the FPKM for the *i*th tissue; and *g*_sum_ is the sum of *N* tissues.

### DNA methylation divergence

DNA methylation divergence was calculated as previously described (Keller and Yi 2014; Kim and Yi 2006). We defined promoter methylation divergence (PMD) as (*M*_P1_ − *M*_P2_) / (*M*_P1_ + *M*_P2_), where *M*_P1_ and *M*_P2_ are the average promoter methylation levels for the first and second gene, respectively. The methylation level was normalized to the overall methylation level of the pair. Similarly, gene body methylation divergence (GMD) was calculated as (*M*_G1_ − *M*_G2_) / (*M*_G1_ + *M*_G2_).

### Specificity index of DNA methylation

The stage-specific patterns of DNA promoter methylation were described using the stage specificity index, which was previously used to assess gene expression (Yanai *et al.* 2005). The specificity index was defined as follows:

$$\mathrm{SMI=}\frac{\sum_{i=\text{1}}^{n}\left(1-m_{i}/m_{\max}\right)}{n-1}$$

where *m_i_* is the methylation in stage *i*, *m*_max_ is the maximum methylation level for a gene across stages, and *n* is the number of stage. Thus, a larger SMI indicates a more stage-specific pattern of DNA methylation. We also calculated the divergence of SMI between duplicate gene pairs as (SMI_1_ − SMI_2_) / (SMI_1_ + SMI_2_).

### Motif-enrichment analysis

We used MEME (Bailey *et al.* 2006) to identify DNA motifs that were distinguished within the promoter regions of consistently methylated *vs.* unmethylated duplicate genes. Considering the large number of sequences, we defined the promoter region as the 1000 bases upstream of the transcription start site (TSS), based on a previous study (Keller and Yi 2014). MEME was used to identify the 10 most significantly different motifs in consistently methylated promoters by generating motif position specific priors (PSPs). Then, MAST (Bailey and Gribskov 1998) was used to calculate the frequency of these motifs in the methylated and unmethylated promoter regions. Finally, we used TOMTOM (Gupta *et al.* 2007) to identify the transcription factor families to which the motifs bound.

### Statistical analysis

In this study, R3.1.1 for windows was used for most statistical analysis. Pearson’s correlation coefficients were used to measure correlations between methylation and evolutionary time. We used the partial correlation with the “ppcor” package in *R* to examine the relative correlation between methylation level and gene expression (Y. Wang *et al.* 2013; Kim and Yi 2006). Multiple testing was corrected by applying the FDR method implemented in *R* (Storey and Tibshirani 2003).

### Data availability

All the corresponding nucleotide and protein sequences of zebrafish were retrieved from Ensembl (http://asia.ensembl.org/index.html). Methylation data for egg, sperm, testis and six stages: 16-cell, 32-cell, 64-cell, 128-cell, 1k-cell and germ ring were obtained from NCBI with accession number PRJNA188516. Available paired end FASTQ sequence files for sperm, egg, 1,000 cells and germ ring were obtained from NCBI with accession number PRJNA188516. RNA-seq data of 16cell, 32cell and 128cell were downloaded with accession number PRJNA127881. RNA-seq data of testis was obtained with SRA number SRR1695730.

## Results

### Generation, quality control, and filter of the duplicate gene dataset

**Figure 1:**
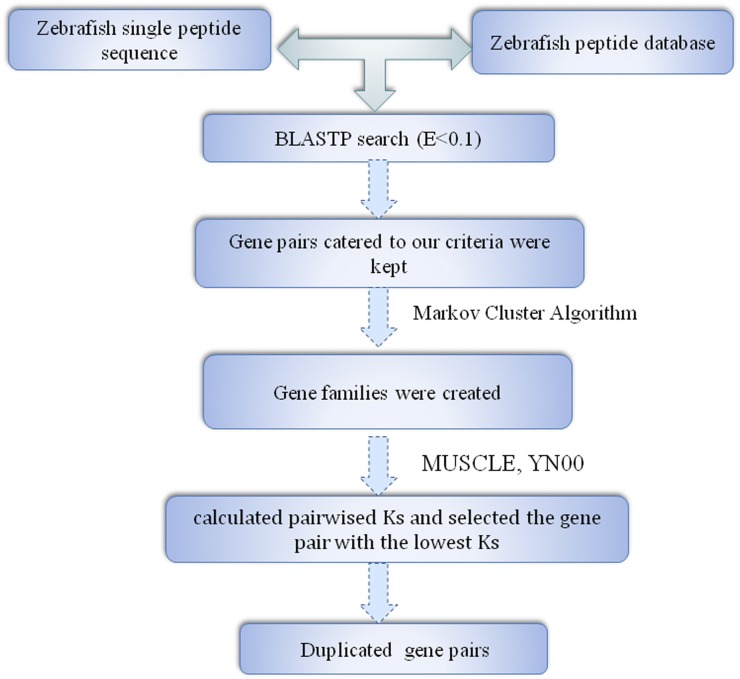
Pipeline of duplicate gene detection in zebrafish.

**Figure 2:**
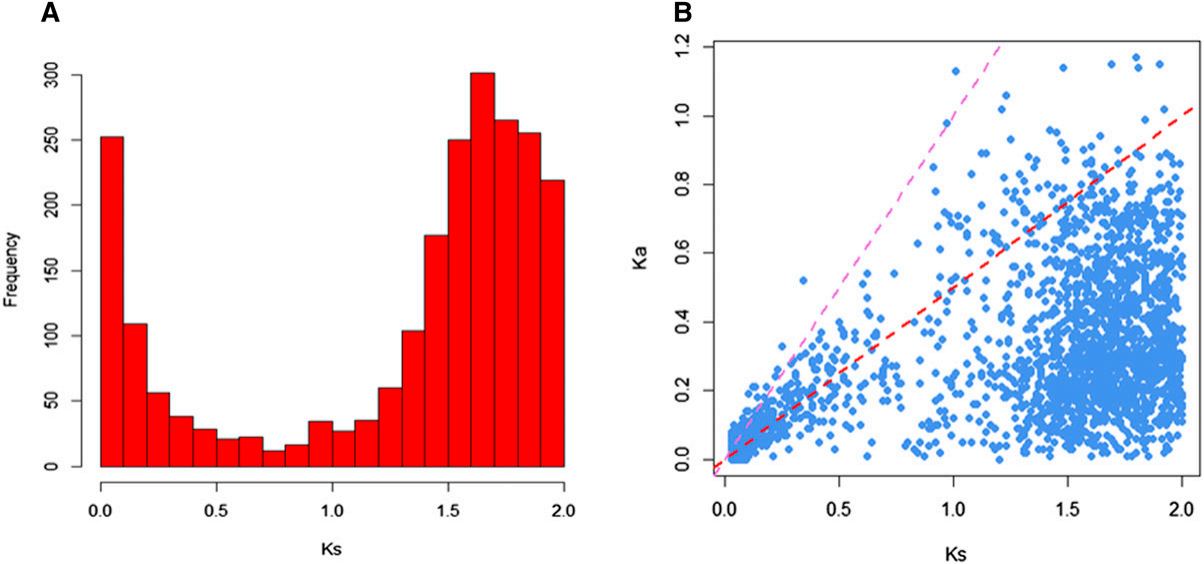
Ks and Ka/Ks distributions. (A) Histogram showing the Ks values for duplicate gene pairs. (B) Distribution of Ka/Ks. The red and purple dashed lines represent Ka/Ks values of 0.5 and 1, respectively.

Using a refined version of a previously published procedure, 2440 pairs of duplicate genes with 0.01 < Ks ≤ 2 were obtained in the zebrafish genome (Figure 1). The distribution of Ks is shown in Figure 2A. The number of duplicate genes tended to increase to a peak value, when the Ks value was 1.6. We hypothesized that the third round WGD (whole genome duplication) may cause this peak. Under a substitution rate of 4.13 × 10^−9^ substitutions per silent site per year (Fu *et al.* 2010), WGD may indicate the birth of duplicate genes ∼387 MYA, which was during the time of the third genome duplication event that occurred in the stem lineage of teleost fish (infraclass Teleostei) after the divergence from nonteleost ray-finned fish (Nakatani *et al.* 2007; Jaillon *et al.* 2004; Hoegg *et al.* 2004; Amores *et al.* 1998,^2011^; Taylor *et al.* 2003; Van de Peer 2003; Meyer and Van de Peer 2005). Such consistency suggests the high quality of our duplicate gene dataset.

The ratio of nonsynonymous substitutions per nonsynonymous site (Ka) to synonymous substitutions per synonymous site (Ks) was calculated for duplicate gene pairs to assess natural selection (Yang 2007); the results are shown in Figure 2B. A likelihood ratio test (LRT) of Ka/Ks (ω) confirmed that the ω of 1851 of 2440 (75.9%) pairs were significantly < 0.5 (Supplemental Material, Table S1, adjusted *P*-value < 0.05), suggesting that both copies of duplicate gene pairs were under purifying selection. That is, genes compiled in our duplicate gene dataset are largely functional.

### Young duplicates tended to be hypermethylated in promoter regions

**Figure 3:**
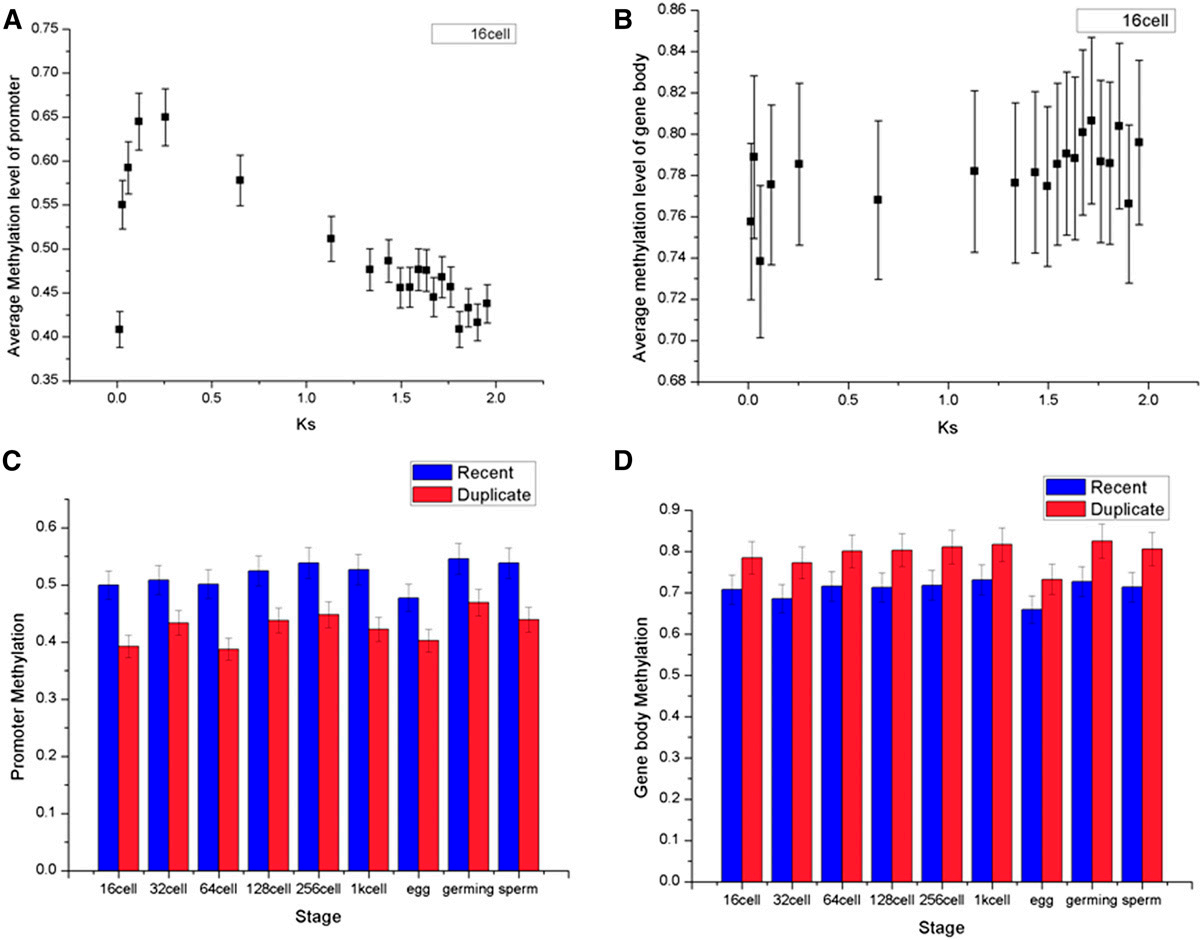
Patterns of relationship between DNA methylation and evolutionary age (Ks). DNA methylation data from the 16-cell stage are divided into 20 groups by Ks. (A) The correlation between the average promoter methylation level and Ks for duplicate gene pairs (Pearson’s *R* = −0.72, *P* < 5.46E−04). (B) Gene body methylation shows a positive correlation with Ks than does promoter methylation (Pearson’s *R* = 0.58, *P* = 7.53E−03). Error bars represent 95% confidence intervals. (C) “Recent” duplicate pairs were single copy in grass carp but duplicates in zebrafish (*n* = 85), which is relative younger than remaining duplicate pairs. Duplicates are significantly (*P* < 0.05) less methylated than young duplicates in promoters. (D) Duplicates are more methylated than young duplicates in gene body.

The average promoter DNA methylation levels were calculated for nine datasets (egg, sperm, testis, 16-cell, 32-cell, 64-cell, 128-cell, 1k-cell, and germ ring), which exhibited a significant negative correlation with evolutionary time measured by Ks. The relationship for a 16-cell embryo (Pearson’s correlation coefficient, *R* = −0.72, *P* < 5.46E−04) is presented in Figure 3A, and the relationship for the other stages are presented in Table S2. However, the average gene body methylation levels exhibited a positive correlation with evolutionary time (Pearson’s correlation coefficient, *R* = 0.58, *P* = 7.53E−03) (Figure 3B for 16-cell embryos, Table S2 for other stages), indicating that the younger gene tended to have a lower promoter methylation level but higher gene body methylation level.

Interestingly, these trends were obvious when we compared methylation levels of “recent” duplicate pairs, which were single copy in grass carp, but duplicates in zebrafish (*n* = 85), to those of duplicate pairs (Table S3). Duplicates are significantly (*P* < 0.05) less methylated than young duplicates in promoters (Figure 3C), while more methylated than young duplicates in gene bodies (Figure 3D).

**Figure 4:**
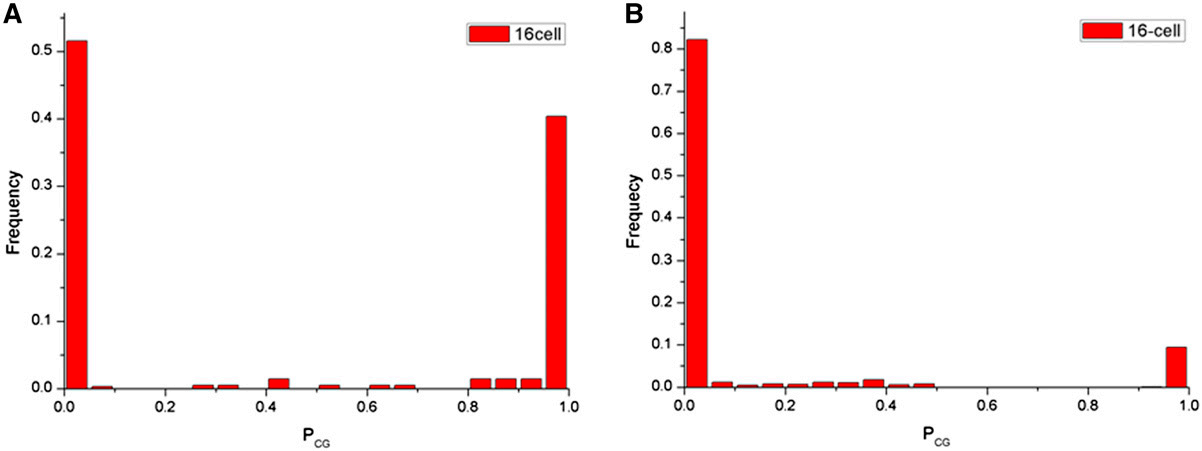
Frequency distribution of P_CG_ as a proxy of CG methylation level. The Lower *P*_CG_ represents higher methylation levels. (A) Frequency promoter-methylated is similar with promoter-unmethylated genes. Methylation data shown are from 16-cell. (B) The majority of genes tend to be gene-body methylated.

To examine this further, we compare the Ks of methylated and unmethylated genes. First, we calculated *P*_CG_ for the promoter region and gene body of each gene, and used the distribution of *P*_CG_ as a proxy for the CG methylation level (see *Materials and Methods*). Only those genes with sufficient CG information (*n*_cg_ ≥ 20), and genes for which 60% of cytosine residues were covered by at least two reads were kept (*n* = 2111) (Takuno and Gaut 2012, 2013). The distribution of P_CG_ of promoter and gene body were both bimodal, indicating that CG methylation is not randomly distributed (Figure 4 and Figure S1). The result showed that Ks ratios were significantly higher in body-methylated genes than in unmethylated genes (Table S4, adjusted *P* value < 2.85E−12). In contrast to that, promoter-methylated genes exhibited lower Ks than unmethylated genes (Table S4, adjusted *P* value < 4.51E−05).

### Methylation divergence of duplicate genes changed along an evolutionary timescale

**Figure 5:**
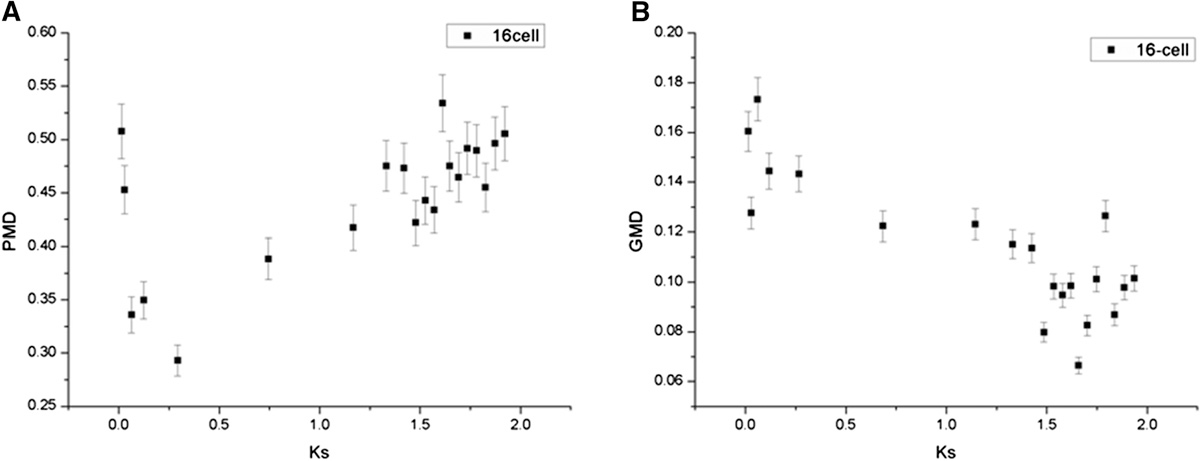
The relationship between methylation divergence and Ks. (A) PMD and Ks are positively correlated (Pearson’s *R* = 0.61, adjusted *P* value < 4.65E−03). Compared with older duplicate gene pairs, the younger pairs tended to exhibit similar levels of promoter methylation. (B) Significantly negative correlation is found between the GMD and Ks (16-cell, Pearson’s *R* = −0.82, adjusted *P* value < 4.65E−03).

To study the dynamics of DNA methylation divergence within duplicate gene pairs, we calculated the relative promoter methylation divergence (PMD) and gene body methylation divergence (GMD) (see *Materials and Methods*). We observed that the PMD and Ks were positively correlated (Figure 5A for 16-cell, Pearson’s *R* = 0.61, adjusted *P* value < 4.65E−03, Figure S2 for other stages). Compared with older duplicate gene pairs, the younger pairs tended to exhibit similar levels of promoter methylation; however, significantly negative correlation was found between the GMD and Ks (Figure 5B, 16-cell, Pearson’s *R* = −0.82, adjusted *P* value < 4.65E−03, Figure S3 for other stages). We also calculated the stage specificity index of DNA promoter methylation (SMI, see *Materials and Methods*), which provided insights into the relative strength of methylation across six early embryo stages (16-cell, 32-cell, 64-cell, 128-cell, 1k-cell, and germ ring). A negative correlation was demonstrated between the relative divergence of SMI and Ks (*R* = −0.33, *P* < 2.12E−15).

### Negative correlation between promoter DNA methylation and gene expression level

**Figure 6:**
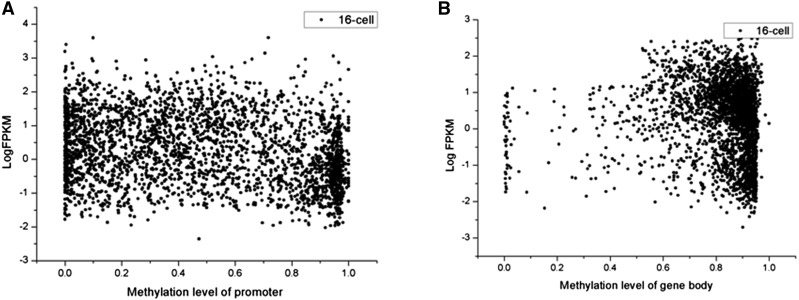
The relationship between methylation and expression level (Log FPKM). (A) Promoters of duplicate genes are heavily methylated initially, and gradually lose DNA methylation cross evolutionary age. (B) In contrast, gene-body DNA methylation exhibits an increasing pattern.

DNA methylation is known to regulate gene expression in mammals and plants, whereby higher levels of promoter methylation silence downstream gene expression (Weber *et al.* 2007; Zemach *et al.* 2010a). We hypothesized that a high level of promoter DNA methylation of duplicate promoters is also associated with low expression of duplicate genes in zebrafish. To explore the relative association of promoter methylation and gene body methylation with expression level, we evaluated partial correlation using the “ppcor” package in *R* (see *Materials and Methods*). Indeed, expression levels were significantly, negatively correlated with promoter methylation levels (Figure 6, *P* < 3.62E−03), whereas no significant correlation was established between the gene body methylation levels and expression levels.

We are wondering whether differential promoter methylation of duplicate gene pairs resulted in different gene expression. Compared with the promoter methylated genes, unmethylated gene exhibited a significant higher expression level (Table S5, two sample *t*-test, adjusted *P* value <1.43E−02).

**Figure 7:**
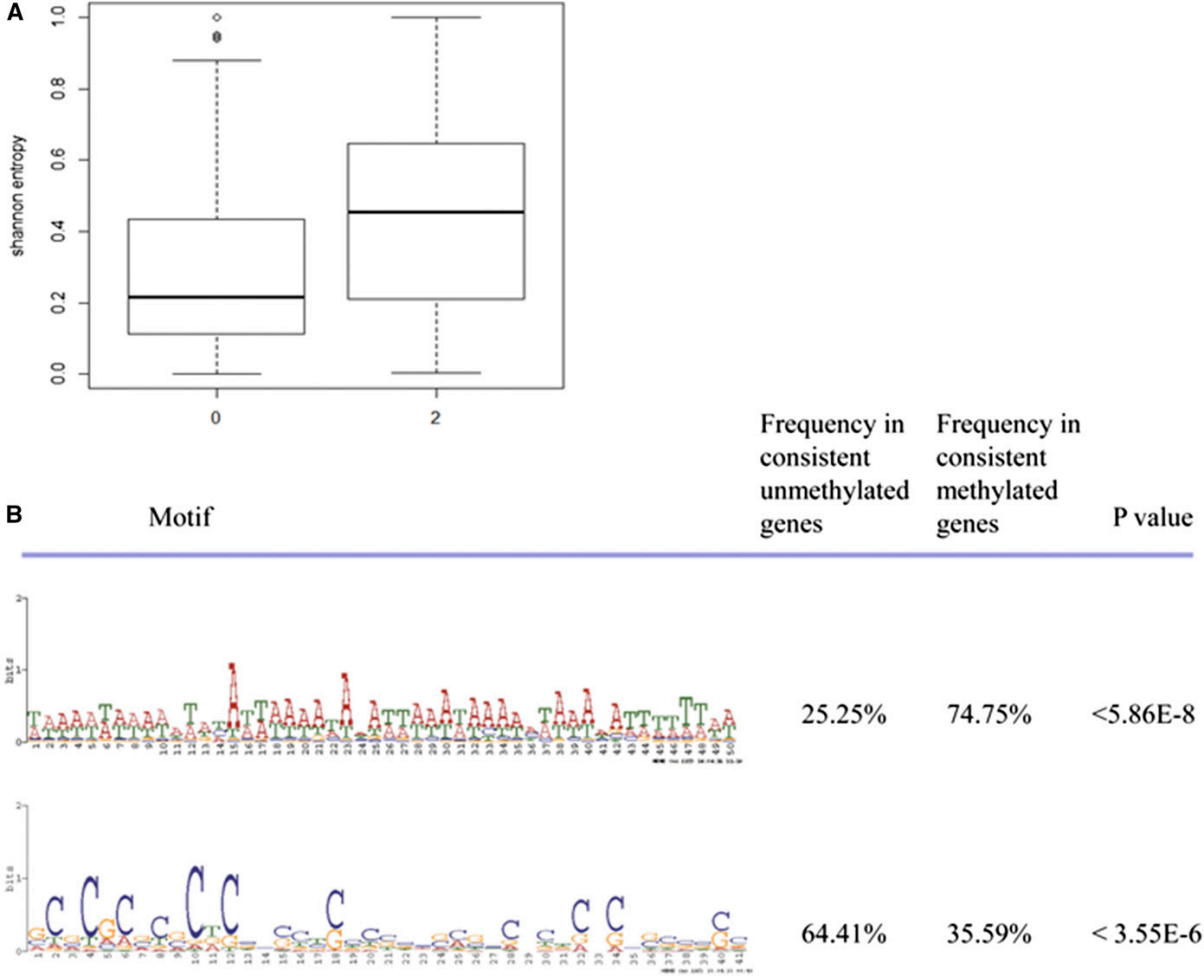
Shannon entropy and motifs comparison between promoter-methylated genes and promoter-unmethylated genes. (A) Promoter-unmethylated genes exhibited significantly lower Shannon entropy. (B) One motif occurred significantly more often in the unmethylated group than in the methylated groups (*P* < 5.86E−8, Fisher’s exact test), whereas another motif occurred significantly less often (*P* < 3.55E−6, Fisher’s exact test).

Moreover, we used Shannon entropy to measure the breadth of expression. The result indicated that the promoter-unmethylated genes exhibited significantly lower Shannon entropy, suggesting a broader expression than promoter-methylated genes (Figure 7A, 4.98E−03).

### Enrichment of specific DNA motifs in consistent methylated promoters

Why do promoter-methylated genes show lower gene expression level? One explanation is that some motifs in the promoter region bind to transcription repressors (Keller and Yi 2014). To test this hypothesis, we identified duplicate gene pairs where one copy was consistently promoter-methylated while the other copy was promoter-unmethylated across the nine samples we studied; 665 duplicate gene pairs fulfilled our criteria (Table S6).

Here, we examined the mechanism that helps distinguish the two promoters. We used a weight matrix finding algorithm (MEME) (Bailey *et al.* 2006, 2010), and a motif search tool (MAST) (Bailey and Gribskov 1998), to identify the 10 most significant motifs that discriminated between methylated and unmethylated groups. One motif occurred significantly more often in the unmethylated group than in the methylated groups (*P* < 5.86E−8, Fisher’s exact test), whereas another motif occurred significantly less often (*P* < 3.55E−6, Fisher’s exact test) (Figure 7B). Interestingly, the motif enriched in the methylated promoters contained the regions binding to Forkhead box D3 (FOXD3) that were previously identified by TOMTOM (Gupta *et al.* 2007). FOXD3 has been reported to function as a transcriptional repressor (Guo *et al.* 2002; Yaklichkin *et al.* 2007). By contrast, the motif enriched in the unmethylated promoters included Sp1 binding sites, which prevent local DNA methylation (Brandeis *et al.* 1994). The difference in the methylation levels of promoters may be explained by the presence of these motifs.

### Body-methylated genes showed longer length than body-unmethylated genes

Previous studies revealed different predictions between body methylation and gene length or exon. For instance, in *Arabidopsis thaliana*, body-methylated genes were significantly longer than unmethylated genes, and have more exons (Takuno and Gaut 2012). Genes with higher C_p_G were significantly longer than those with lower C_p_G (Zeng and Yi 2010).

**Figure 8:**
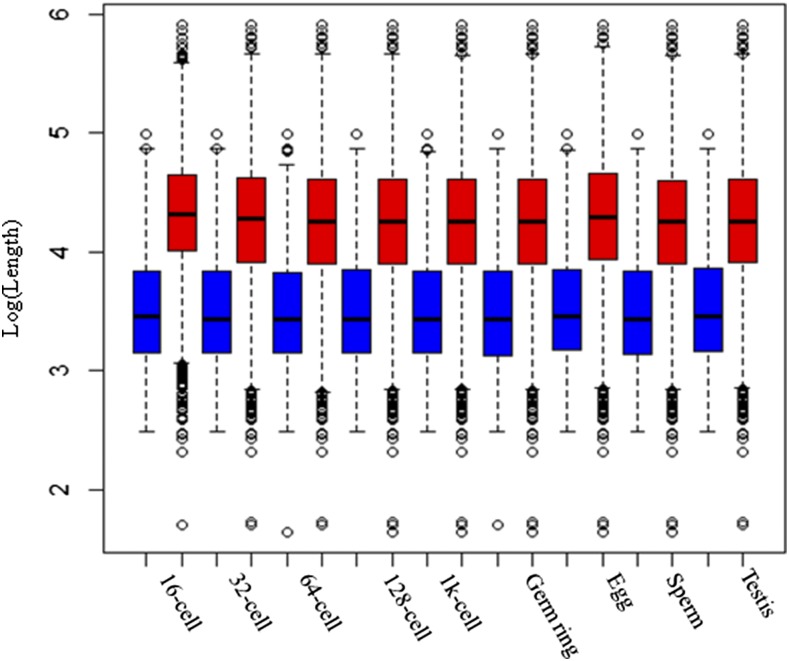
The distribution of gene length. The blue box represents body-unmethylated genes, and the red box represents body-methylated genes. Mean length of the body-methylated genes was significantly longer than the mean length of unmethylated genes (adjusted *P* value < 2.2e−16).

In this study, our data also support these predictions. We found the mean length of the body-methylated genes was significantly longer than the mean length of unmethylated genes (37,582.78 *vs.* 7655.52 bp for 16 cell, Table S7 for other stages, two sample *t*-test, adjusted *P* value < 2.2e−16). Figure 8 shows the distribution of gene length.

### Methylation divergence is associated with functional divergence

Furthermore, we attempted to assess the relationship between functional divergence and methylation in zebrafish.

First, we used the DAVID Functional Annotation tool to assess enrichment of gene ontology (GO) terms. Functional annotation clustering results of the 2440 pairs of duplicate genes revealed that the majority of these genes were enriched in the following biological process categories: immunoglobulin-like, hexose catabolic process and protein catabolic process, and glycolysis. These genes may be involved in degradation, including the breakdown of sugar and proteins; post-translational modification; and transcription factor activity.

Second, we used Interproscan (Jones *et al.* 2014) to predict the functional domains of the duplicate genes (Table S8). In the 2440 pairs, 181 pairs had no functional domains in either paralog. This result could potentially be explained by the fact that the paralog might not have been fully studied. For the remaining 2259 pairs, of which at least one copy had domain annotation, both copies in 414 pairs had different functional domains. Within 626 duplicate gene pairs that showed consistent promoter divergence and had functional domains, 169 pairs showed function divergence. However, within 1633 duplicate gene pairs that did not show consistent promoter divergence, only 245 pairs exhibited function divergence. The two-sample test for equality of proportions with continuity correction was implemented in *R* 3.1.2 and a significant difference (*P* < 2.2e−16) was found. Our results may indicate that methylation divergence may be associated with functional divergence.

## Discussion

DNA-mediated duplication, that is, the duplication of chromosomal segments containing genes, has been widely studied (Lynch and Conery 2000; Taylor *et al.* 2003). DNA duplication is a critical source of genetic innovation that plays a key role in evolution (Assis and Bachtrog 2013). After duplication, genes are subject to a series of processes, including expression and functional divergence. In our study, we performed a comparative analysis of the association of epigenetic modification, such as DNA methylation, with the evolutionary divergence of duplicate genes.

### The promoter regions of younger duplicate genes are generally methylated

It was known that most newly born genes may degrade to pseudogenes. Epigenetic silencing of duplication might play an important role in shifting the loss *vs.* gain equilibrium (Rodin and Riggs 2003). In our study, we noticed that the promoter regions of younger duplicate genes tended to be methylated, whereas those old duplicates were generally unmethylated (Figure 3, C and D). Remarkably, a similar pattern has also been found in humans (Keller and Yi 2014). Thus, it is possible that newly duplicated genes have the same expression pattern, and are epigenetically silenced in a tissue- or stage-complementary manner, which protects each of the duplicates from “pseudogenization” (Rodin and Riggs 2003).

### Evolutionary conservation of gene body methylation

According to previous studies, gene bodies consistently exhibit higher levels of methylation compared with promoters (Jjingo *et al.* 2012). Moreover, our study showed that gene body methylation was significantly correlated with Ks (Pearson’s *R* = 0.58, Figure 3B). In humans, gene-body DNA methylation and Ks are negatively correlated, but this correlation is extremely weak (Pearson’s *R* = −0.06) (Keller and Yi 2014). Gene body methylation is reportedly conserved between plants and animals (Zemach *et al.* 2010b; Sarda *et al.* 2012; Zeng and Yi 2010), whereas a study in rice suggests that gene body divergence is associated with Ks (Y. Wang *et al.* 2013). However, GMD in zebrafish did not exhibit a discernible relationship with Ks. This result suggested that the epigenetic modification of the gene body might be subject to Ks, whereas GMD between duplicate genes was relatively conserved.

Differential gene body DNA methylation covaries with gene length between duplicate genes. It has been hypothesized that body methylation has a functional role, perhaps in transcriptional accuracy or splicing efficiency. Consistently, we demonstrated that methylated duplicate genes have longer gene length.

### Methylation of promoters, rather than that of gene bodies, is associated with transcription levels

In mammals, DNA methylation of promoter regions is a repressive mark, and depresses gene expression (Zemach *et al.* 2010b; Elango and Yi 2008; Boyes and Bird 1992; Shen *et al.* 2007), whereas intragenic methylation is associated with gene expression by controlling the expression from alternative promoter regions (Maunakea *et al.* 2010). Additionally, in plant genomes, including *A. thaliana* (Arabidopsis), *Oryza sativa* (rice), *Populus trichocarpa* (poplar), and *Chlamydomonas reinhardtii* (green algae), gene-body methylation tends to influence transcription level (Feng *et al.* 2010; Zemach *et al.* 2010b; Takuno and Gaut 2013). Even a single CpG within a transcription-factor-binding site potentially influences gene regulation (Ziller *et al.* 2013). Indeed, we demonstrated that promoter methylation was significantly correlated with gene expression (Figure 6), whereas no significant correlation was observed between gene body methylation and expression level in zebrafish. Meanwhile, promoter-unmethylated genes exhibited significantly lower Shannon entropy, suggesting a broader expression than promoter-methylated genes. The relationship between DNA methylation and transcription level potentially varies between taxa. Consistent with previous studies that raised the notion of “expression reduction model” and “gene dosage balance,” our results indicated that heavy promoter methylation following the duplication event may offset the expression level to avoid detrimental mutations (Rodin and Riggs 2003; Chang and Liao 2012).

Remarkably, the motif-enrichment results revealed that the Forkhead-related transcriptional regulator FOXD3 is present at a significantly higher frequency within the methylated promoters. A previous study of gene expression has suggested that FOXD3 is involved in a negative autoregulatory mechanism (Chiang *et al.* 2001; Dottori *et al.* 2001). Moreover, our study demonstrated that promoter methylation divergence of duplicate genes also affects gene expression, indicating that epigenetic divergence potentially influences transcription levels. Comparative genome analysis regarding duplicate genes supports the hypothesis that differential DNA methylation and epigenetic changes play a role in protecting duplicate genes from pseudogenization (Rodin *et al.* 2005; Cortese *et al.* 2008).

### Genetic influences on DNA methylation variation

Previous study has proved that DNA methylation variation is influenced by genetic and epigenetic changes that are often stably inherited and can influence the expression of nearby genes (Eichten *et al.* 2013). In this study, we also tried to assess the influence of nucleotide divergence, especially at C nucleotides and CpG di-nucleotides between duplicates, on DNA methylation in zebrafish. First, we tried to identify duplicates that display differential methylation but have little to no sequence variation. These could be more likely candidates of true epigenetic variation. However, since zebrafish went through the third genome duplication 320–400 MYA, duplicate genes diverge greatly in sequence. Then, we carefully assessed the *C* content of promoter regions between duplicate pairs with a customized Perl script. On the one hand, we narrowed down a set of duplicate genes that have diverged greatly in their *C* content. We found that 356 duplicate pairs (Table S9) show differential methylation, possibly because the *C* content was different, which was caused by divergence at the nucleotide level.

Our study gives strong support to the idea that epigenetic divergence of duplicate genes affects gene expression and functional divergence of duplicate genes.

## 

## Supplementary Material

Supplemental Material
