# Supplementary material for: Divergent DNA Methylation Provides Insights into the Evolution of Duplicate Genes in Zebrafish
Source: G3 (Bethesda). 2016 Sep 19;6(11):3581–91. doi: 10.1534/g3.116.032243 (PMC5100857; doi:10.1534/g3.116.032243)
Supplement: Supplemental Material [file supp_6_11_3581__index.html]

Divergent DNA Methylation Provides Insights into the Evolution of Duplicate Genes in Zebrafish — Supplemental Material 

# Divergent DNA Methylation Provides Insights into the Evolution of Duplicate Genes in Zebrafish

## Supplemental Material for Zhong, *et al*, 2016

**Files in this Data Supplement:**

- Figure S1 - Frequency distribution of PCG as a proxy of CG methylation level. The Lower PCG represents higher methylation levels. (.tif, 133 KB)
- Figure S2 - Promoter methylation divergence (PMD) show significant negative correlation with Ks. (.tif, 126 KB)
- Figure S3 - Gene body methylation divergence (GMD) show significant positive correlation with Ks. (.tif, 121 KB)
- Table S1 - The Ks and ω of 2440pairs duplicate genes. LRT (likelihood ratio test) was carried out using PAML. The false discovery rates (FDR) were controlled using the Benjamini-Hochberg method with an FDR of 5%. (.xlsx, 261 KB)
- Table S2 - Correlation between methylation level and Ks. (.xlsx, 11 KB)
- Table S3 - Information of Recent duplicate genes. (.xlsx, 35 KB)
- Table S4 - Comparison of Ks between methylated and unmethylated genes. (.xlsx, 11 KB)
- Table S5 - Average FPKM of genes. (.xlsx, 11 KB)
- Table S6 - Duplicate genes with promoter methylation divergence. (.xlsx, 33 KB)
- Table S7 - Average gene length comparison between body-methylated and body-unmethylated genes. (.xlsx, 11 KB)
- Table S8 - Interproscan result of duplicate genes. (.xlsx, 121 KB)
- Table S9 - Gene ID with promoter methylation divergence influnced by nucleotide variation. (.xlsx, 24 KB)
