# Supplementary figures and images for: Divergent DNA Methylation Provides Insights into the Evolution of Duplicate Genes in Zebrafish
Source: G3 (Bethesda). 2016 Sep 19;6(11):3581–91. doi: 10.1534/g3.116.032243 (PMC5100857; doi:10.1534/g3.116.032243)

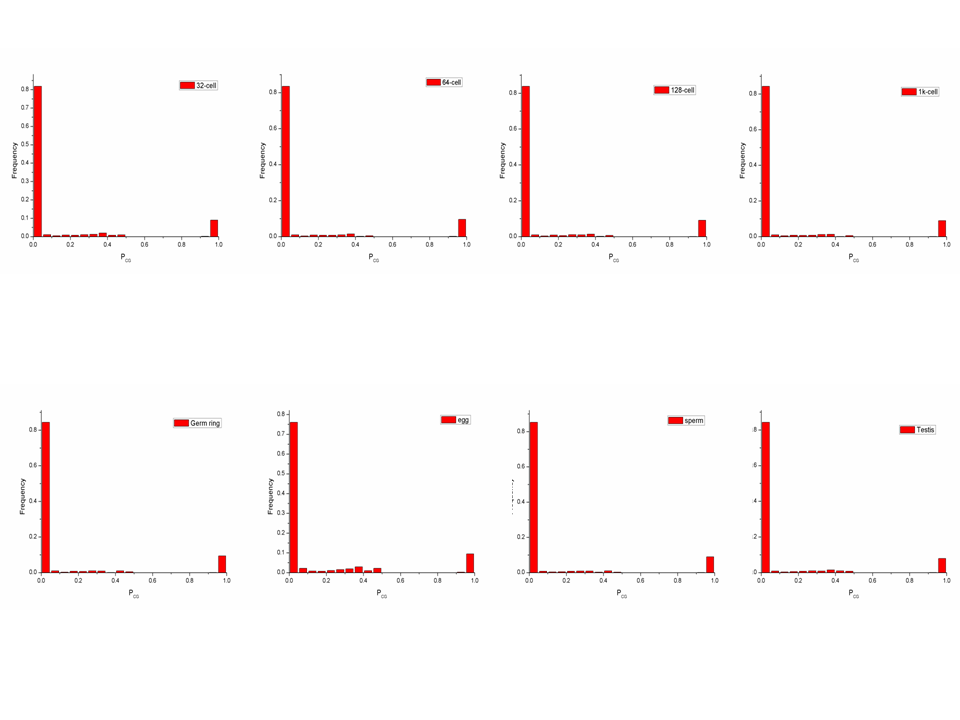

Supplement: Supplemental Material [file supp_g3.116.032243_FigureS1.tif]

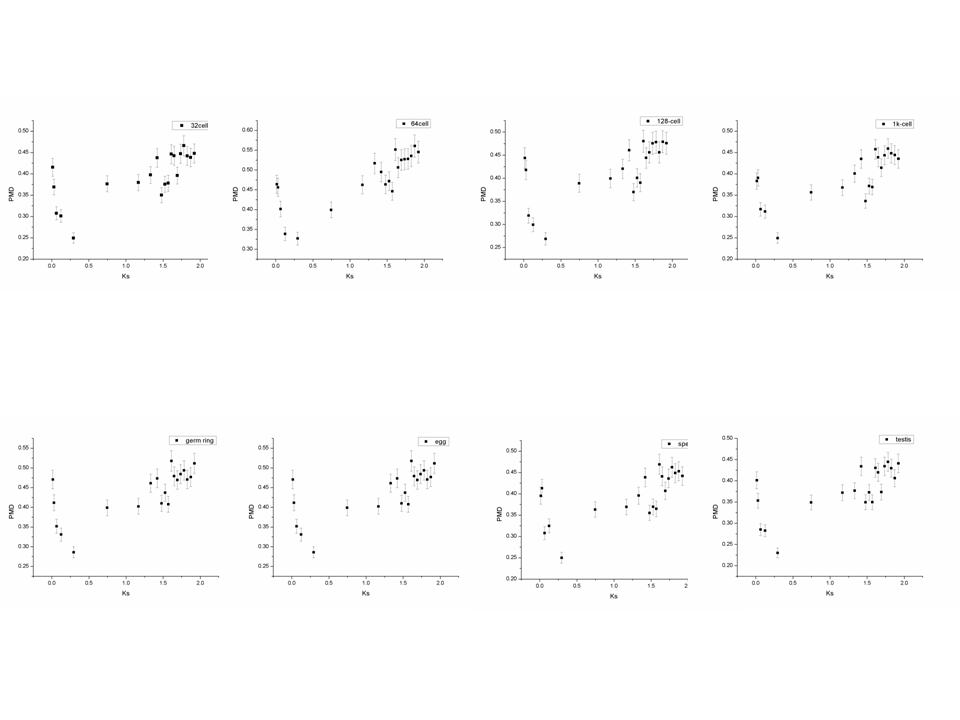

Supplement: Supplemental Material [file supp_g3.116.032243_FigureS2.tif]

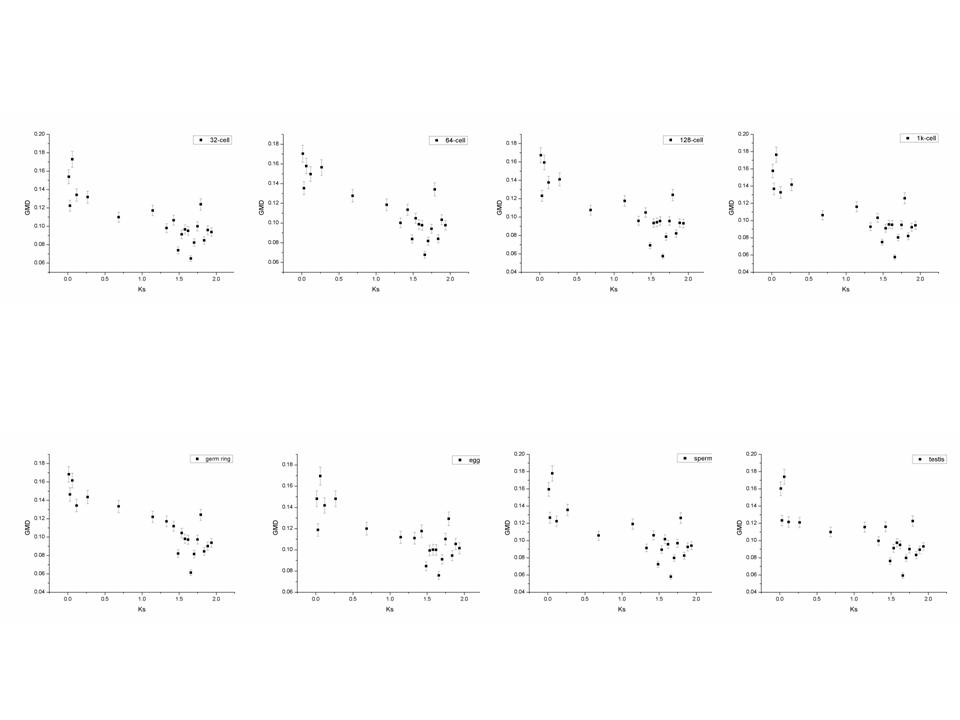

Supplement: Supplemental Material [file supp_g3.116.032243_FigureS3.tif]
